# Succinic acid inhibits hepatocyte regenerative signaling via STAT3 SH2-domain engagement

**DOI:** 10.1186/s43556-026-00535-z

**Published:** 2026-08-08

**Authors:** Yiyuan Chen, Nan Xu, Hao Chen, Zhaofeng Xiao, Hengkai Zhu, Zhengxing Lian, Zhikun Liu, Zhe Yang, Caide Lu, Shusen Zheng, Qiang Wei, Shuai Wang, Xiao Xu

**Affiliations:** 1 Zhejiang University School of Medicine, Hangzhou, 310058 China; 2 Hepatopancreatobiliary Center, School of Clinical Medicine, Beijing Tsinghua Changgung Hospital, Tsinghua University, Beijing, 102218 China; 3 The Fourth School of Clinical Medicine, Zhejiang Chinese Medical University, Hangzhou, 310053 China; 4 Department of Hepatobiliary and Pancreatic Surgery, Key Laboratory of Artificial Organs and Computational Medicine in Zhejiang Province, Shulan (Hangzhou) Hospital, Shulan International Medical College, Zhejiang Shuren University, Hangzhou, 310022 China; 5 Key Laboratory of Integrated Oncology and Intelligent Medicine of Zhejiang Province, Hangzhou First People’s Hospital, Hangzhou, 310006 China; 6 School of Clinical Medicine, Hangzhou Medical College, Hangzhou, 311399 China; 7 Department of Hepatopancreatobiliary Surgery, Ningbo Medical Center Lihuili Hospital, Ningbo, 315000 China; 8 Shulan (Hangzhou) Hospital Affiliated to Zhejiang Shuren University, Shulan International Medical College, Hangzhou, 310000 China; 9 Hepatobiliary Center and Transplantation Center, The First Affiliated Hospital of Nanjing Medical University, Nanjing, 210000 China

**Keywords:** Split liver transplantation, Liver regeneration, Succinic acid, TCA cycle

## Abstract

The restoration of liver mass after partial hepatectomy (PH) and split liver transplantation (SLT) relies on efficient liver regeneration, a process highly susceptible to metabolic dysregulation. Although remodeling of the tricarboxylic acid (TCA) cycle is commonly observed during liver injury and regeneration, the specific role of succinic acid remains unclear. Here, we integrated radiomics, transcriptomics, metabolomics, and functional assays to systematically evaluate the association between succinic acid and liver regeneration. Multi-omics analysis of 20 SLT patients demonstrated that elevated succinic acid levels were strongly associated with impaired regenerative outcomes, and weighted gene co-expression network analysis identified the TCA cycle as a key regeneration-associated metabolic module. In a 70% PH mouse model, succinic acid administration was associated with reduced survival and decreased hepatocyte proliferation, consistent with impaired liver regeneration. Mechanistically, succinic acid exhibited distinct effects in different cell types: in macrophages, it enhanced inflammatory activation and increased interleukin-6 (IL-6) secretion, whereas in hepatocytes it reduced IL-6-induced STAT3 phosphorylation and downstream regenerative signaling. Furthermore, molecular docking, molecular dynamics simulations, and surface plasmon resonance assays supported an interaction between succinic acid and the STAT3 SH2 domain, providing structural evidence consistent with modulation of STAT3 signaling. Collectively, these findings suggest that succinic acid may function as an immunometabolic regulator that contributes to impaired liver regeneration by altering macrophage-derived IL-6 signaling and hepatocyte STAT3 activation. These findings also support further investigation of the succinic acid–STAT3 axis as a potential therapeutic target for promoting liver regeneration following hepatic injury or resection.

**Supplementary Information:**

The online version contains supplementary material available at 10.1186/s43556-026-00535-z.

## Introduction

Liver diseases represent a major global health burden, accounting for approximately 2 million deaths annually worldwide, including deaths caused by cirrhosis, liver failure, and hepatocellular carcinoma [1, 2]. Since the first successful liver transplantation in 1963 and its subsequent standardization in the 1970s, liver transplantation has remained the definitive treatment for end-stage liver disease, achieving a five-year survival rate of 72%–84% [3]. The emergence of split liver transplantation (SLT) has alleviated the problem of donor liver shortage [4]; however, it is technically challenging and often complicated by small-for-size syndrome [5]. Liver regeneration is an essential physiological process that restores hepatic function after injury, which relies on the liver’s intrinsic regenerative capacity [6, 7].

Hepatocyte reprogramming requires immune activation to initiate proliferation during the early stage [8, 9], while hepatocytes are the major proliferating cells during the entire liver regeneration process [10, 11]. Hepatocytes adapt their metabolic programs to meet the elevated demands of cellular proliferation [12]. Regeneration requires enhanced mitochondrial oxidative phosphorylation [13], whereas early-stage hepatocytes rely on glycolysis to rapidly produce ATP and biosynthetic intermediates such as acetyl-CoA and NADPH [13, 14]. For instance, AMPK inhibits mTORC1 to prevent excessive regeneration, while mTORC1 later promotes protein synthesis [15]. Metabolites such as S-adenosylmethionine and α-ketoglutarate (α-KG) modulate histone methylation and acetylation, thereby regulating regeneration-related gene expression [16, 17]. Bile acids also serve as signaling molecules, efficiently regulating liver function through the Takeda G-protein-coupled receptor 5 in macrophages, and play key roles in lipid and glucose homeostasis [18]. Other signaling pathways, including Interleukin-6 (IL-6) [19], tumor necrosis factor-alpha (TNF-α) [20], Wnt/beta-catenin [21], and Hedgehog ligands [22], also regulate liver regeneration.

The tricarboxylic acid (TCA) cycle supports hepatocyte proliferation during liver regeneration through the production of energy and anabolic metabolites. Recent studies have identified succinic acid, a key TCA-cycle metabolite, as an important regulator of inflammation and metabolic signaling [23]. SUCNR1 is highly expressed in macrophages, where its activation promotes inflammatory responses, and has also been implicated in liver injury and metabolic homeostasis [24, 25].

Despite increasing evidence linking metabolic remodeling to liver regeneration, whether TCA-cycle metabolites modulate hepatocyte regenerative signaling remains unclear. Using integrated multi-omics analyses in SLT patients and functional studies in a 70% partial hepatectomy (PH) mouse model, we found that elevated succinic acid was associated with impaired hepatocyte proliferation and liver regeneration. Mechanistic analyses further supported an interaction between succinic acid and the STAT3 SH2 domain, which was associated with reduced IL-6–STAT3 signaling, suggesting that succinic acid may function as an immunometabolism regulator during liver regeneration.

Our study investigated the potential role of succinic acid in liver regeneration. Using integrated multi-omics analyses and mechanistic experiments, we found that succinic acid was associated with reduced hepatocyte proliferation and impaired liver regeneration. Our data further support an interaction between succinic acid and the STAT3 SH2 domain that may contribute to attenuation of IL-6–STAT3 signaling.

## Results

### Evaluate the liver regeneration rate using radiomics

**Fig. 1:**
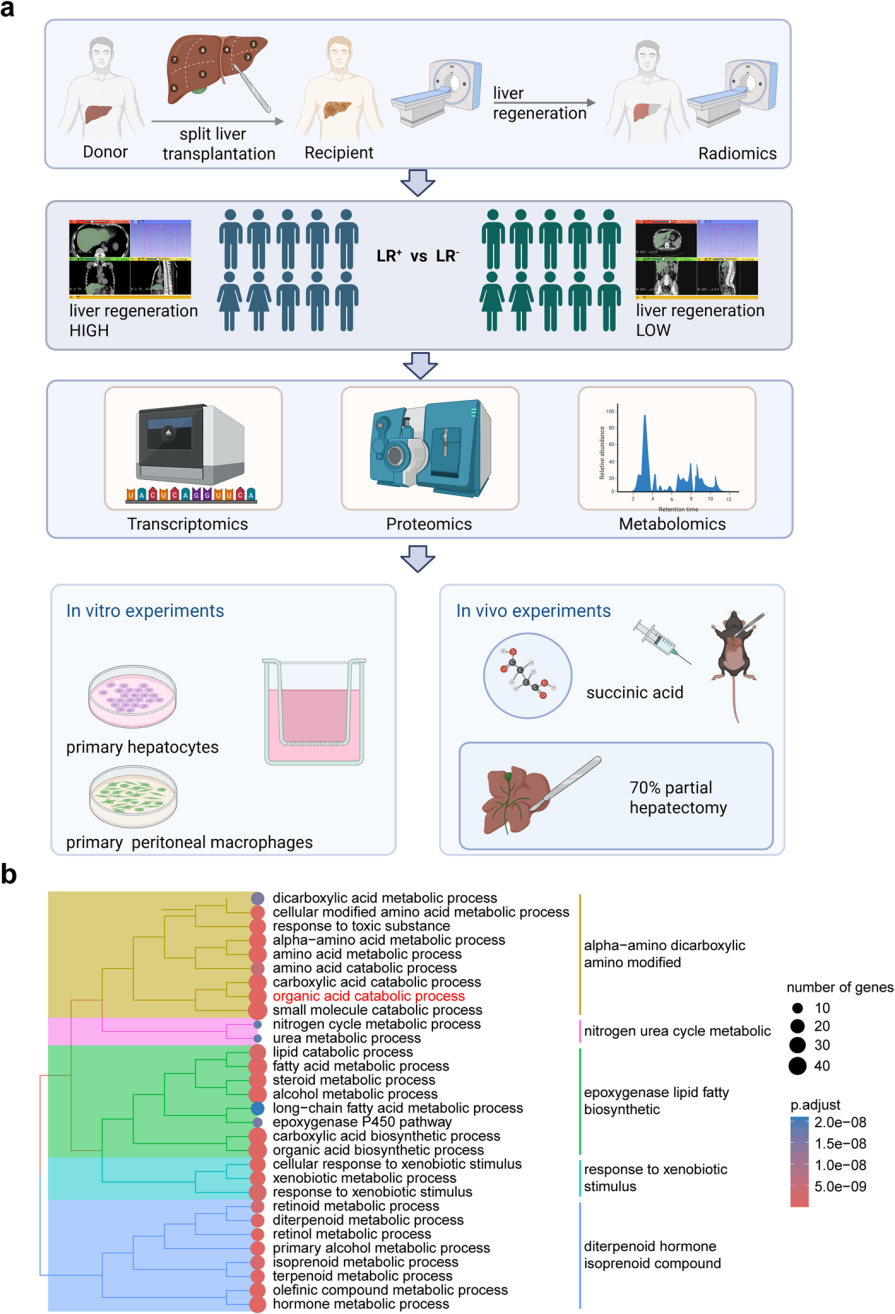
Multi-omics profiling identifies succinic acid accumulation associated with poor liver regeneration. **a** Schematic overview of the study design. Patients undergoing split liver transplantation were monitored for liver regeneration (LR) by imaging-based volumetry and stratified into high (LR^+^) and low (LR^−^) groups. Multi-omics profiling was performed to identify metabolic determinants of regenerative capacity. **b** Gene Ontology (GO) enrichment analysis of LR^+^ versus LR^−^ groups. Circle size represents the number of enriched genes, while the color scale denotes adjusted *p*-values

A retrospective cohort of 55 patients undergoing split liver transplantation was analyzed to investigate variations in liver regeneration. After excluding patients with severe underlying disorders, 20 patients were included for further analysis, and their baseline characteristics are presented in Table 1. We used 3D Slicer to reconstruct the patients’ livers from their CT images. Standard liver volume (SLV) was calculated based on liver weight, body weight, and height. We divided these 20 patients into two groups (liver regeneration high: LR^+^ and liver regeneration low: LR^−^) based on their liver regeneration rate. To investigate the liver metabolite landscape in response to liver resection, we used quasi-targeted metabolomics and transcriptomics approaches to identify key factors regulating liver regeneration (Fig. 1a). Initially, we aimed to identify genes and pathways that were significantly associated with liver regeneration. A total of 13,680 differentially expressed genes were identified; subsequently, KEGG enrichment analysis was conducted on these differentially expressed genes. Pathway enrichment analysis identified marked enrichment of metabolism-related pathways, including organic acid catabolism, amino acid metabolic processes, and steroid metabolism (Fig. 1b).

**Table 1:** Baseline characteristics of 20 patients

		**LR**^**+**^** (*N* = 10)**	**LR**^**−**^**(*N* = 10)**	***P***
Recipients sex	Male	7	8	> 0.99
	Female	3	2	
Donor sex	Male	10	10	> 0.99
	Female	0	0	
Recipients age		51.20 ± 3.31	58.40 ± 3.12	0.13
Donor age		44.10 ± 11.56	45.00 ± 16.45	0.09
Diagnose	Non-Cancer	4	5	> 0.99
	Cancer	6	5	
BMI		23.44 ± 1.59	24.42 ± 1.07	0.61
Diabetes	-	7	8	> 0.99
	+	3	2	
Hypertension	-	7	7	> 0.99
	+	3	3	
HBV	-	4	3	> 0.99
	+	6	7	
Cigarette	-	7	8	> 0.99
	+	3	2	
Alcohol	-	8	5	0.35
	+	2	5	
AFP	-	7	7	> 0.99
	+	3	3	
Pre low GV/SLV	-	7	8	> 0.99
	+	3	2	
Increased GV/SLV		0.49 ± 0.05	0.06 ± 0.05	****

**** = *P* < 0.0001, Mean ± SD

### Weighted gene co-expression network analysis identified regeneration-related modules

**Fig. 2:**
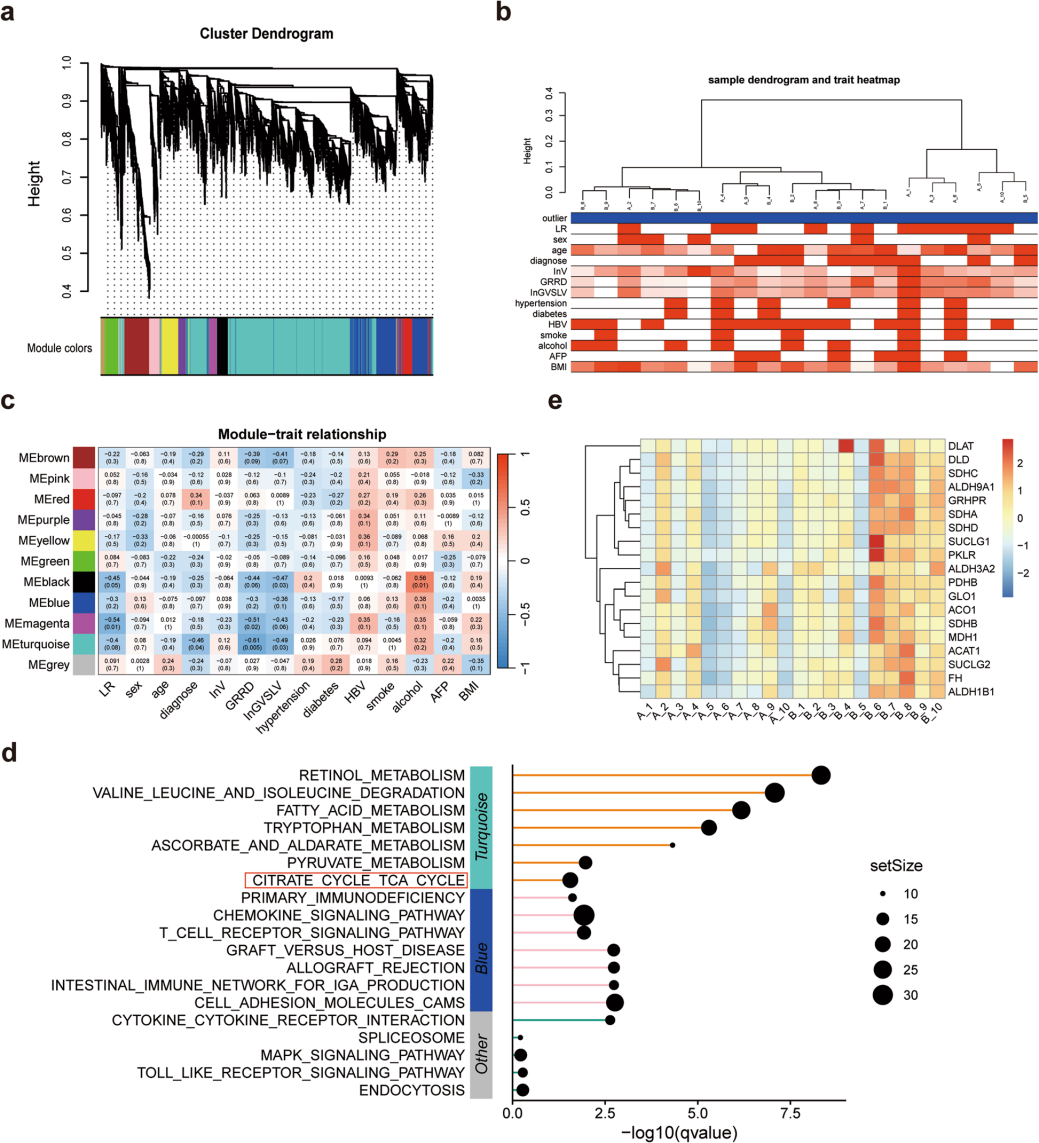
Weighted Gene Co-expression Network Analysis (WGCNA) Identifies Modules Associated with Liver Regeneration. **a** Hierarchical clustering dendrogram of genes based on topological overlap, with modules assigned by dynamic tree cutting and labeled by color. The top is the gene dendrogram, and the bottom is the gene modules with different colors. A total of 11 modules were identified in this study (The grey module represents genes that do not belong to any modules). **b** Sample dendrogram and trait heatmap illustrating the relationship between patient samples and clinical features. **c** Module–trait correlation heatmap. Each cell shows the correlation coefficient (top) and *p*-value (bottom) between module eigengenes and clinical traits. Color scale reflects correlation strength, with red indicating positive and blue indicating negative associations. **d** KEGG pathway enrichment of modules significantly correlated with LR. Enriched pathways include the TCA cycle, pyruvate metabolism, fatty acid metabolism, and immune-related signaling. Circle size represents gene set size, and the x-axis denotes enrichment significance (–log10 q value). **e** Heatmap of representative hub genes within the TCA cycle, highlighting metabolic enzymes and regulatory factors with coordinated expression across patient samples

We next performed weighted gene co-expression network analysis (WGCNA) on liver samples from patients with diverse clinical features to identify gene modules associated with liver regeneration. Based on gene expression similarity, hierarchical clustering analysis identified multiple distinct co-expression modules (Fig. 2a). Sample clustering combined with clinical trait heatmap analysis illustrated the distribution of patient characteristics, including liver regeneration rate, age, BMI, diabetes, and hypertension (Fig. 2b). Further module–trait correlation analysis demonstrated significant associations between specific gene modules and regenerative phenotypes (Fig. 2c). Among these, the magenta, black, and turquoise modules exhibited the strongest correlations with liver regeneration. In addition, the purple module was predominantly associated with HBV infection, whereas the gray module showed the strongest association with alcohol consumption. To further characterize the biological functions of each module, KEGG pathway enrichment analysis was performed for all modules containing sufficient genes and visualized as a bubble plot. Notably, the turquoise module, which negatively correlated with liver regeneration, was significantly enriched in metabolic pathways, including the TCA cycle, fatty acid metabolism, pyruvate metabolism, and tryptophan metabolism (Fig. 2d). To further examine metabolic alterations within the regeneration-associated module, we analyzed the expression of TCA cycle–related genes. As shown in Fig. 2e, multiple enzymes involved in the TCA cycle exhibited coordinated expression changes between regeneration groups, including *SUCLG*, *SDHA*, *SDHB*, *SDHD*, *FH*, *MDH1* and *DLAT*, supporting the notion that TCA cycle remodeling is closely associated with impaired liver regeneration.

Chronic liver diseases, including HBV infection and Liver metabolism diseases, such as diabetes, also influence liver regeneration [26, 27]. We therefore further stratified patients according to diabetes and HBV status. In the diabetes subgroup, differential gene expression analysis revealed enrichment of pathways related to glycine, serine and threonine metabolism, valine, leucine and isoleucine degradation, focal adhesion, EGFR tyrosine kinase inhibitor resistance, and MAPK signaling, suggesting that metabolic disorders are associated with coordinated alterations in both metabolic and immune-regulatory pathways during liver regeneration (Fig. S1a). Similarly, HBV-associated transcriptional changes were enriched in pathways related to hepatitis B, chemokine signaling, NF-κB signaling, TNF signaling, oxidative phosphorylation, and PPAR signaling, indicating that chronic HBV infection may influence liver regeneration through combined inflammatory, immune, and metabolic mechanisms (Fig. S1b).

### Transcriptomic-metabolite correlation analysis

**Fig. 3:**
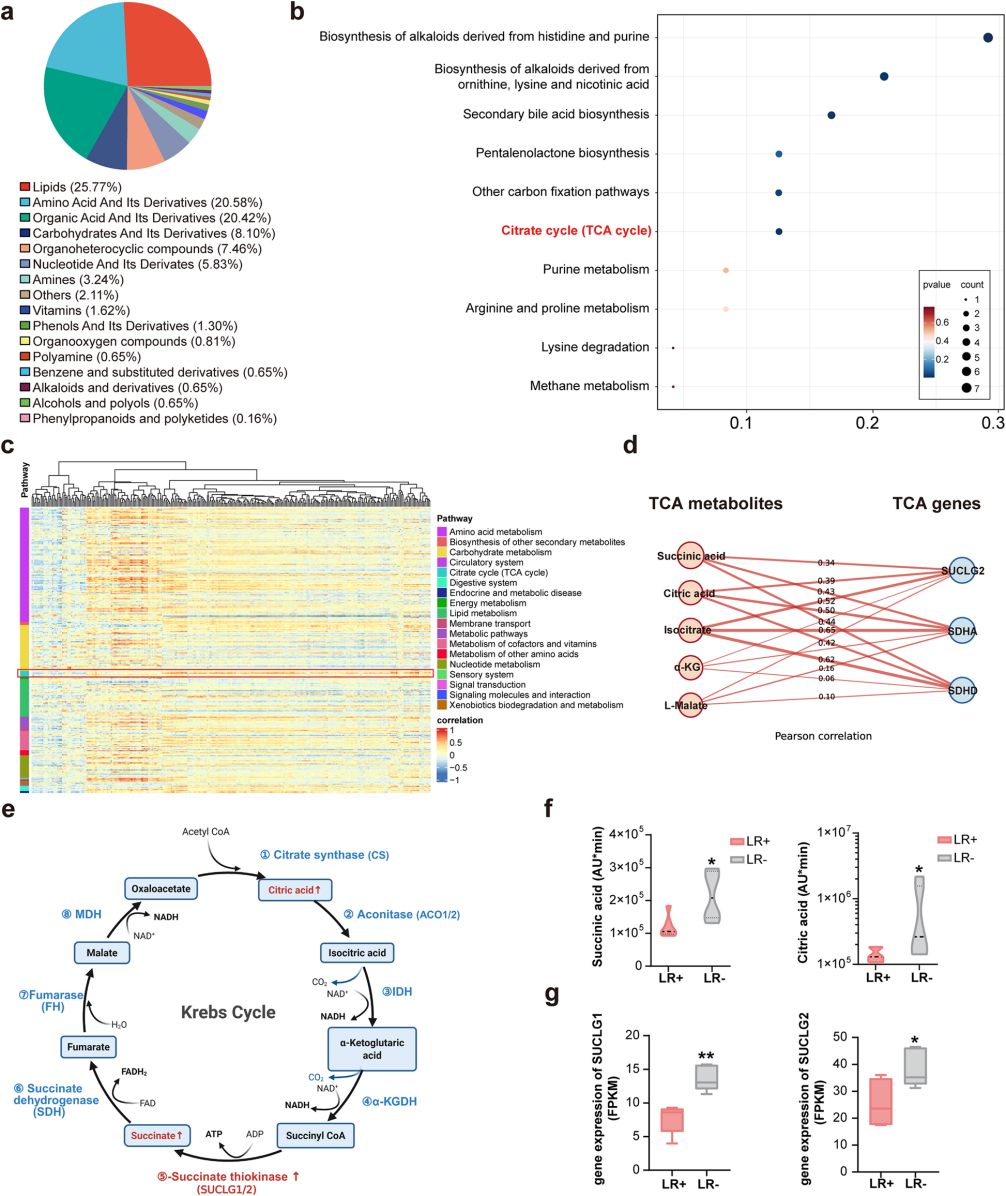
Transcriptomic–metabolomic analysis identifies TCA cycle-associated metabolic alterations during impaired liver regeneration. **a** Pie chart showing the classification of identified metabolites according to KEGG annotation. **b** KEGG pathway enrichment analysis indicates significantly enriched metabolic pathways. **c** Visualization of the protein-metabolite correlations using a heatmap demonstrated distinct patterns of associations between individual proteins and members of specific classes of metabolites. Individual metabolites were ordered according to RefMet class along the y-axis, and proteins were ordered using a hierarchical cluster analysis along the x-axis. The magnitude and directionality of the correlation coefficient for each protein-metabolite association are depicted by color, as indicated in the legend. **d** Correlation network showing associations between representative TCA cycle metabolites and TCA-related genes. Red nodes indicate TCA metabolites, and blue nodes indicate TCA genes. Edge labels indicate Pearson correlation coefficients between metabolite abundance and gene expression levels. **e** Schematic representation of the enzymatic steps of the TCA cycle and the upregulated metabolites. **f** Violin plots showing relative abundances of representative metabolites in the TCA cycle. **g** Boxplots showing tissue-specific concentrations of TCA cycle intermediates in liver samples

To further investigate metabolic alterations associated with impaired liver regeneration, we performed metabolomic profiling of liver tissues from LR⁺ and LR⁻ patients. KEGG annotation demonstrated that the identified metabolites were predominantly enriched in lipids, amino acids and their derivatives, and organic acids (Fig. 3a). Differential metabolite pathway enrichment analysis revealed significant alterations in multiple metabolic pathways, with the TCA cycle remaining prominently enriched, consistent with our transcriptomic findings (Fig. 3b).

Integrated transcriptomic–metabolomic correlation analysis further demonstrated coordinated associations between metabolic alterations and transcriptional programs across multiple pathways (Fig. 3c). To further investigate the relationship between TCA cycle remodeling and changes in gene expression, we constructed a multi-omics integration network. Correlation analysis revealed associations between TCA metabolites and key TCA enzyme genes, including Succinate-CoA ligase GDP-forming subunit beta (*SUCLG2*), Succinate dehydrogenase complex flavoprotein subunit A (*SDHA*), and *SDHD* (Fig. 3d). We next summarized the enzymatic reactions of the TCA cycle and highlighted key regulatory enzymes involved in succinate metabolism (Fig. 3e). Quantitative analysis of representative TCA cycle intermediates demonstrated significant accumulation of succinic acid and citric acid in LR⁻ liver tissues compared with LR⁺ samples (Fig. 3f), suggesting dysregulated TCA cycle flux during impaired liver regeneration. Consistently, transcriptomic analysis revealed significant dysregulation of TCA cycle enzyme genes in LR⁻ liver tissues, with *SUCLG1* and *SUCLG2* exhibiting marked upregulation (Fig. 3g), which was consistent with the accumulation of succinic acid observed in metabolomic profiling. Reanalysis of published spatial transcriptomic data further supported these findings [28]. Heatmap analysis showed a significant upregulation of TCA cycle–related metabolic genes after partial PH (Fig. S2a–b). These genes were predominantly enriched in periportal hepatocytes and cyclin hepatocyte clusters on day 7 post-PH (Fig. S2c).

### The TCA cycle metabolite, succinic acid, inhibits liver regeneration

**Fig. 4:**
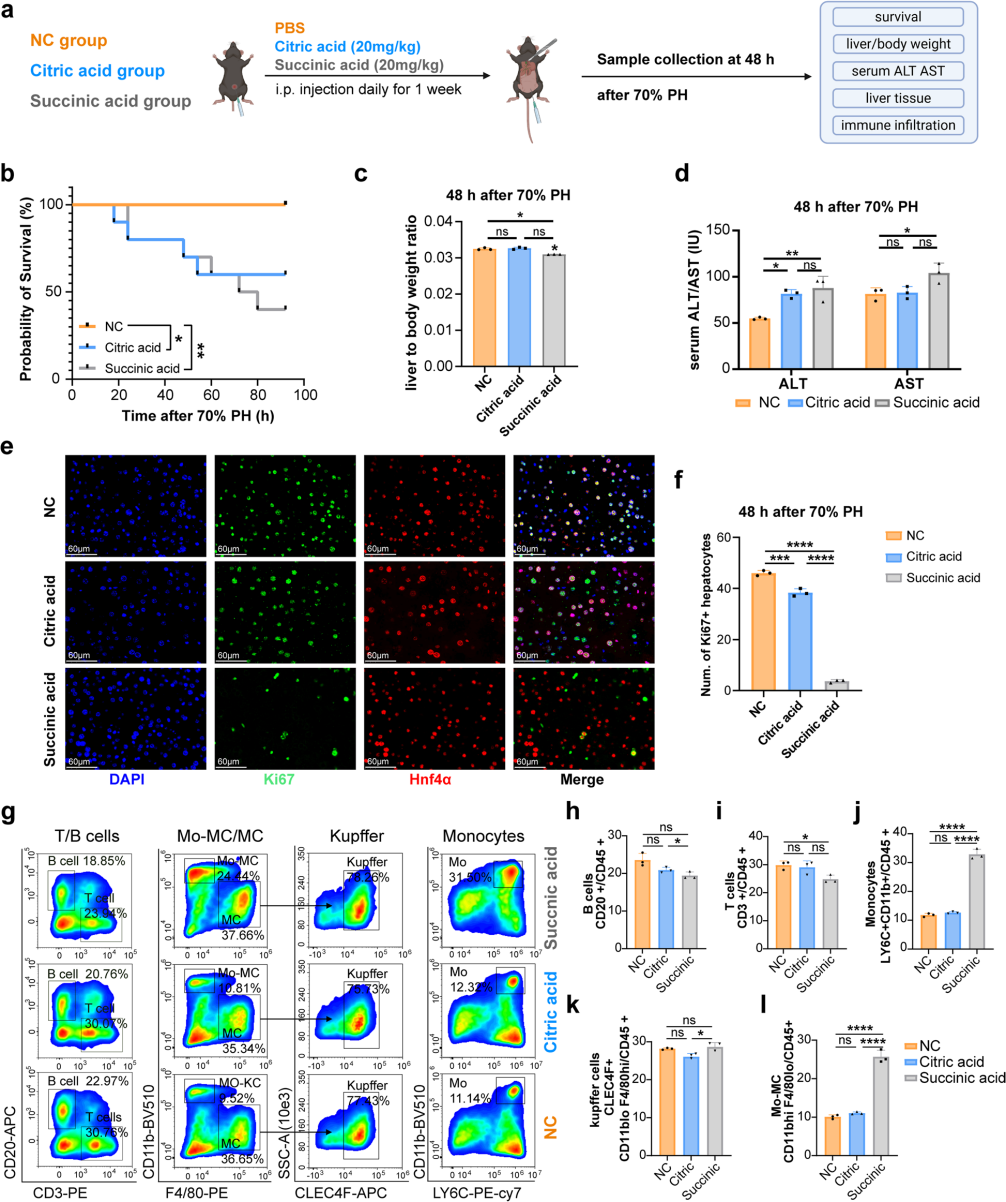
Succinic acid administration is associated with reduced liver regeneration and immune cell alterations after partial hepatectomy. **a** Schematic illustration of the experimental design. Mice received daily intraperitoneal injections of PBS, citric acid, or succinic acid (20 mg/kg) for 1 week before 70% partial hepatectomy (PH). Samples were collected 48 h after PH for liver-to-body weight ratio measurement, serum biochemical analysis, histological examination, and immune cell profiling. **b** A separate cohort of mice was used for survival analysis and monitored for 96 h after PH. Kaplan–Meier survival analysis of mice following 70% partial hepatectomy (PH) with saline control, citric acid, or succinic acid treatment. Statistical significance was analyzed using the log-rank test. (*n* = 10). **c** The liver-to-body weight ratio at 48 h after PH. Liver-to-body weight ratio = liver weight/body weight (g/g). (*n* = 3). **d** Serum levels of transaminase-alanine aminotransferase (ALT) and aspartate aminotransferase AST) at 48 h after 70% PH. (*n* = 3). **e** Immunofluorescence staining of liver sections for Ki67 (proliferation marker, green), Hnf4α (hepatocyte marker, red), and DAPI (blue). Scale bars, 60 μm. (*n* = 3). **f** Quantification of Ki67^+^ hepatocytes in liver sections shown in (e). (*n* = 3). **g** Representative flow cytometry plots showing hepatic immune cell populations. T cells (CD45 + CD3 +), B cells (CD45^+^CD20^+^), Monocytes (CD45^+^CD11b^+^ Ly6C^+^), Kupffer cells (CD45^+^CD11b^lowF4/80^hiCLEC4F^+^), and monocyte-derived macrophages (Mo-MC: CD45^+^CD11b^hiF4/80^low) in total immune cells at 48 h after PH. (*n* = 3). **h-l** Quantification of hepatic B cells, T cells, monocytes, Kupffer cells, and monocyte-derived macrophages in the indicated groups. (*n* = 3). Data are presented as mean ± SEM. Statistical significance was determined using one-way ANOVA followed by multiple-comparison testing. ns, not significant; *P < 0.05; **P < 0.01; ***P < 0.001; ****P < 0.0001

Integrated transcriptomic and metabolomic analyses identified succinic acid and citric acid as key dysregulated TCA cycle metabolites. We therefore evaluated their effects on liver regeneration using a 70% PH mouse model. Mice received succinic acid or citric acid (20 mg/kg/day) for 1 week before PH (Fig. 4a). Administration of succinic acid or citric acid exerted distinct effects on post-hepatectomy survival, serum ALT and AST levels, and the liver-to-body weight ratio (Fig. 4b–d). In particular, succinic acid markedly suppressed the expression of regeneration-related genes, including *MKi67, Pcna, Ccnd1, Cdk1, and Sox9* (Fig. S2d). Succinic acid administration markedly impaired hepatocyte proliferation, as evidenced by a reduced proportion of Ki67-positive hepatocytes among Hnf4α-expressing cells (Fig. 4e–f). We also detected variations in the immune cells using flow cytometry. Succinic acid treatment alters hepatic immune cell composition after 70% partial hepatectomy (Fig. 4g). T cells (CD45^+^CD3^+^) and B cells (CD45^+^CD20^+^) showed only minor changes following succinic acid treatment (Fig. 4h–i). In contrast, hepatic monocytes (CD45^+^CD11b^+^Ly6c^+^) markedly increased following succinic acid treatment (Fig. 4j), accompanied by a significant expansion of monocyte-derived macrophages (CD45^+^F4/80^low^CD11b^hi^), whereas resident Kupffer cells (CD45^+^F4/80^hi^CD11b^low^CLEC4F^+^) remained largely unchanged (Fig. 4k–l).

Taken together, these findings suggest that succinic acid plays a more prominent role in regulating liver regeneration.

### Hepatic succinic acid accumulation is associated with impaired liver regeneration after partial hepatectomy

**Fig. 5:**
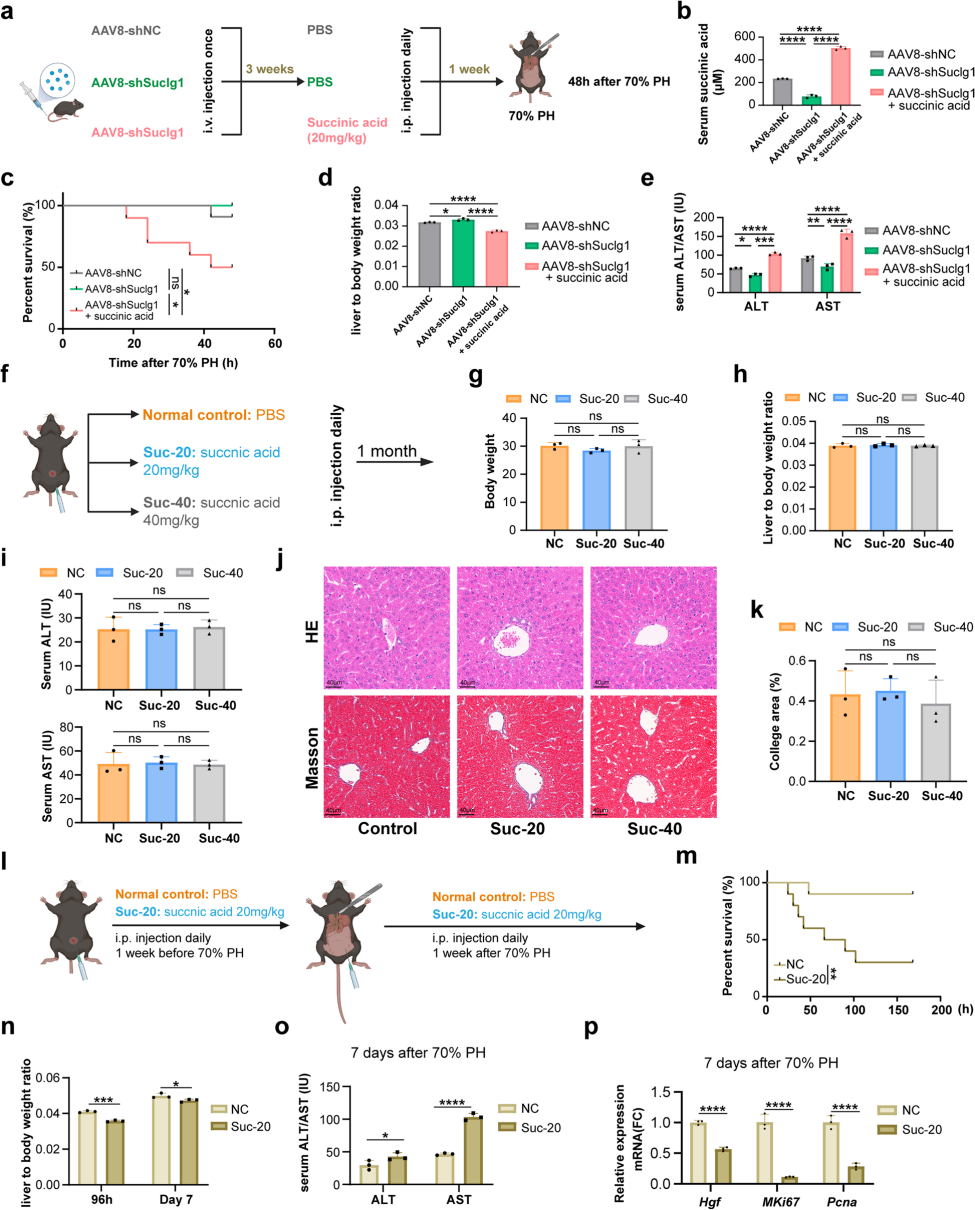
Endogenous succinic acid levels regulate liver regeneration after partial hepatectomy, and evaluation of succinic acid safety and long-term effects. **a** Schematic illustration of the experimental design. Mice received a single tail vein injection of AAV8-shNC or AAV8-shSuclg1. Three weeks later, mice were treated with PBS or succinic acid via daily intraperitoneal injections for 1 week before undergoing 70% partial hepatectomy (PH). Samples were collected 48 h after PH. **b** Serum succinic acid levels in the indicated groups. (*n* = 3). **c** Kaplan–Meier survival curves of mice after 70% PH in the indicated groups. Statistical significance was analyzed using the log-rank test. (*n* = 10). **d** Liver-to-body weight ratio at 48 h after 70% PH. (*n* = 3). **e** Serum alanine aminotransferase (ALT) and aspartate aminotransferase (AST) levels at 48 h after PH. (*n* = 3). **f** Schematic illustration of the succinic acid safety evaluation in normal mice. Mice received daily intraperitoneal injections of PBS or succinic acid (20 or 40 mg/kg) for 1 month. **g** Body weight of mice after long-term succinic acid administration. (*n* = 3). **h** Liver-to-body weight ratio after long-term succinic acid administration. (*n* = 3). **i** Serum ALT and AST levels after long-term succinic acid administration. (*n* = 3). **j** Representative hematoxylin and eosin (H&E) staining and Masson staining of liver tissues from the indicated groups after long-term succinic acid treatment. Scale bars, 40 μm. (*n* = 3). **k** Quantification of collagen-positive area in Masson-stained liver sections shown in (j). (*n* = 3). **l** Schematic illustration of succinic acid administration before and after 70% PH. Mice received daily intraperitoneal injections of PBS or succinic acid (20 mg/kg) for 1 week before PH and continued treatment for 1 week after surgery. **m** Kaplan–Meier survival curves of mice after 70% PH under the indicated treatment conditions. Statistical significance was analyzed using the log-rank test. (*n* = 10). **n** Liver-to-body weight ratio at 96 h and 7 days after PH. (*n* = 3). **o** Serum ALT and AST levels at 7 days after PH. (*n* = 3). **p** qRT–PCR analysis of liver regeneration-associated genes, including *Hgf*, *Ki67*, and *Pcna*, at 7 days after PH. (*n* = 3). Data are presented as mean ± SEM. Statistical significance was determined using one-way ANOVA or two-way ANOVA followed by multiple-comparison testing unless otherwise indicated. ns, not significant; *P < 0.05; **P < 0.01; ***P < 0.001; ****P < 0.0001

To determine whether succinic acid accumulation contributes to impaired liver regeneration, we used AAV8-mediated shRNA knockdown of *Suclg1* in the liver to reduce hepatic succinic acid levels, followed by 70% PH to assess regenerative capacity (Fig. 5a). AAV8-sh*Suclg1* significantly decreased serum succinic acid levels compared with the control group (Fig. 5b). Under these conditions, AAV8-sh*Suclg1* improved survival, reduced serum ALT and AST levels, and increased the liver-to-body weight ratio after PH. In contrast, exogenous succinic acid supplementation partially reversed these improvements and was associated with reduced regenerative outcomes (Fig. 5c–e).

Given previous reports linking succinic acid to liver fibrosis [29], we assessed the potential hepatotoxicity of succinic acid at the dose used in this study. Long-term administration of succinic acid for one month had no apparent impact on body weight, liver-to-body weight ratio, or serum liver enzyme levels. Masson staining (hematoxylin and eosin [HE]) indicates no evidence of liver injury or fibrosis (Fig. 5f–k). Furthermore, prolonged succinic acid administration after 70% PH was also associated with poorer liver regenerative outcomes, as indicated by reduced survival, decreased liver-to-body weight ratio, elevated serum ALT and AST levels, and downregulation of regeneration-associated genes, including *Hgf*, *MKi67*, and *Pcna* (Fig. 5l–p). Notably, the inhibitory effects were more pronounced at the early stage than at later time points, suggesting that succinic acid primarily interferes with the initiation phase of liver regeneration.

Collectively, these findings indicate that elevated succinic acid levels alone do not cause detectable injury under physiological conditions, but sustained succinic acid accumulation after 70% PH was associated with reduced regenerative capacity.

### Succinic acid differentially affects macrophage inflammatory activation and hepatocyte STAT3 signaling

**Fig. 6:**
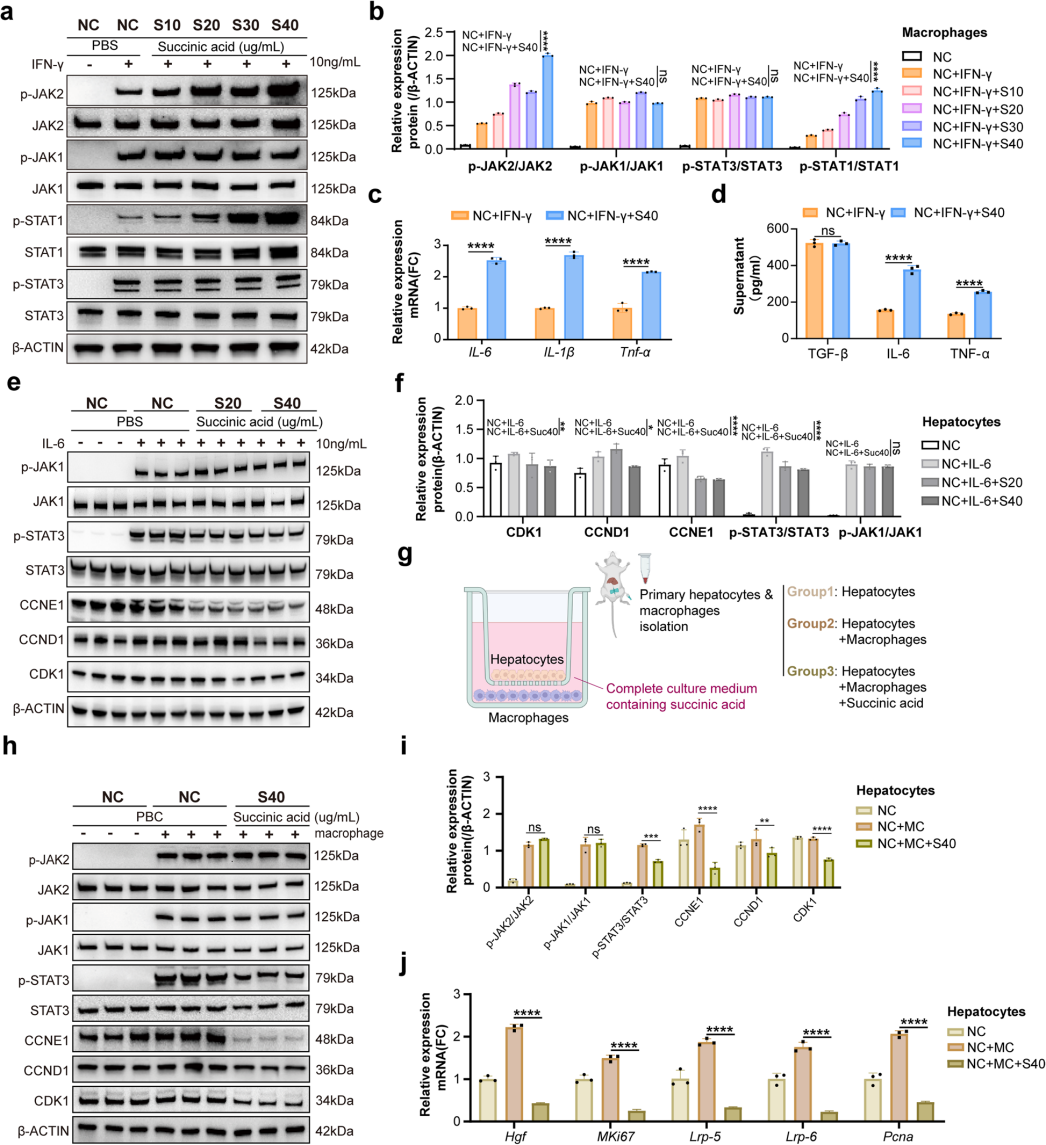
Succinic acid differentially affects macrophage activation and hepatocyte STAT3 signaling. **a** Macrophages were treated with succinic acid (10–40ug/mL) or PBS as a control for 24 h and stimulated with 10 ng/mL IFN-γ for 30 min. JAK1, p-JAK1, JAK2, p-JAK2, STAT1, p-STAT1 (Tyr701), STAT3, p-STAT3, and β-actin protein levels were detected using western blotting. β-actin served as the loading control. (JAK1/2: Janus kinase 1/2; p-JAK1/2: phospho-JAK1/2; STAT1/3: Signal transducer and activator of transcription 1/3; p-STAT1 (Tyr701): phospho-STAT1/3). (*n* = 3). **b** Quantification of the relative phosphorylation levels of JAK1, JAK2, STAT1, and STAT3 shown in (A). Protein expression was normalized to the corresponding total protein levels. **c** qRT–PCR analysis of inflammatory cytokine gene expression, including *IL-6*, *IL-1β*, and *Tnf-α*, in macrophages treated with IFN-γ with or without succinic acid (40 μg/mL). (*n* = 3). **d** ELISA quantification of TGF-β, IL-6, and TNF-α levels in macrophage culture supernatants under the indicated conditions. (*n* = 3). **e** Primary hepatocytes were treated with Succinic acid (40 μg/mL) or PBS as a control for 24 h and stimulated with 10 ng/mL IL-6 for 30 min. JAK1, p-JAK1, STAT3, p-STAT3, CCNE1, CCND1, CDK1, and β-actin protein levels were detected using western blotting. Protein levels were normalized to β-actin expression in each sample. (CCNE1: Cyclin E1; CCND1: Cyclin D1; CDK1: Cyclin-dependent kinase 1). (*n* = 3). **f** Quantification of protein expression levels shown in (e). Relative phosphorylation or protein expression was normalized to the corresponding controls. (*n* = 3). **g** Schematic illustration of the hepatocyte–macrophage co-culture system. Primary hepatocytes and macrophages were isolated and co-cultured in complete medium with or without succinic acid treatment. (*n* = 3). **h** Representative western blot analysis of IL-6–STAT3 signaling and cell cycle-related proteins in hepatocytes cultured alone or co-cultured with macrophages in the presence or absence of succinic acid (40 μg/mL). (*n* = 3). **i** Quantification of protein expression levels shown in (h). Relative phosphorylation or protein expression was normalized to β-actin or the corresponding total protein levels. (*n* = 3). **j** qRT–PCR analysis of hepatocyte proliferation-associated genes, including *Hgf*, *MKi67, Lrp5*, *Lrp6*, and *Pcna,* in the indicated co-culture conditions. (*n* = 3). Data are presented as mean ± SEM. Statistical significance was determined using one-way ANOVA or two-way ANOVA followed by multiple-comparison testing. ns, not significant; *P < 0.05; **P < 0.01; ***P < 0.001; ****P < 0.0001

Based on flow cytometry results showing a marked increase in monocyte-derived macrophages in succinate-treated PH mice (Fig. 4l), we further investigated in vitro experiments to investigate how succinic acid regulates liver regeneration and whether macrophages are involved in this process. In primary mouse macrophages, succinic acid treatment significantly enhanced Ifn-γ–induced inflammatory signaling (Fig. 6a–b), accompanied by increased mRNA expression of *Tnf*, *IL-1b*, and *IL-6* (Fig. 6c), as well as elevated secretion of IL-6 and TNF-α into the culture supernatant (Fig. 6d). Given the distinct cellular responses during liver regeneration, we subsequently examined whether succinic acid also directly affects primary hepatocytes. Succinic acid treatment alone was associated with reduced hepatocyte proliferation and regeneration-related signaling (Fig. 6e–f). As SUCNR1 is highly expressed in macrophages, we examined its involvement in succinate-induced macrophage activation. SUCNR1 inhibition partially reduced inflammatory cytokine expression and secretion, as well as STAT1 phosphorylation in succinate-treated macrophages (Fig. S3a–e), suggesting that SUCNR1 may contribute, at least in part, to succinate-induced macrophage activation.

Although macrophage activation and elevated IL-6 secretion have been reported to promote liver regeneration [30], our in vivo and in vitro results showed that succinic acid impairs the regeneration process. These findings prompted us to examine whether succinate exerts distinct effects on macrophages and hepatocytes. To mimic the hepatic microenvironment in vitro, we established a Transwell co-culture system containing macrophages and hepatocytes (Fig. 6g). Under co-culture conditions, succinic acid reduced the expression of regeneration-associated proteins. It was associated with reduced STAT3 phosphorylation in hepatocytes (Fig. 6h–i) and was accompanied by downregulation of regeneration-associated gene expression in hepatocytes (Fig. 6j).

To determine whether macrophage-derived IL-6 contributes to Succinic acid-mediated inhibition of hepatocyte regeneration, conditioned medium (CM) from Succinic acid-treated macrophages was transferred to primary hepatocytes with or without IL-6 neutralizing antibody (Fig. S3f). CM from Succinic acid-treated macrophages promoted hepatocyte STAT3 activation and proliferative responses compared with control CM. Addition of an IL-6-neutralizing antibody to Succinic acid-CM markedly reduced STAT3 phosphorylation and the expression of proliferation-associated markers in hepatocytes, indicating that macrophage-derived IL-6 mediates a pro-regenerative paracrine effect (Fig. S3g–h).

Collectively, these findings suggest that succinic acid has distinct effects on macrophages and hepatocytes. In macrophages, succinic acid enhances inflammatory activation and IL-6 production in a partially SUCNR1-dependent manner, whereas in hepatocytes, succinate is associated with reduced IL-6-induced STAT3 signaling under our experimental conditions.

### Succinic acid interacts with the STAT3 SH2 domain and is associated with reduced liver regeneration

**Fig. 7:**
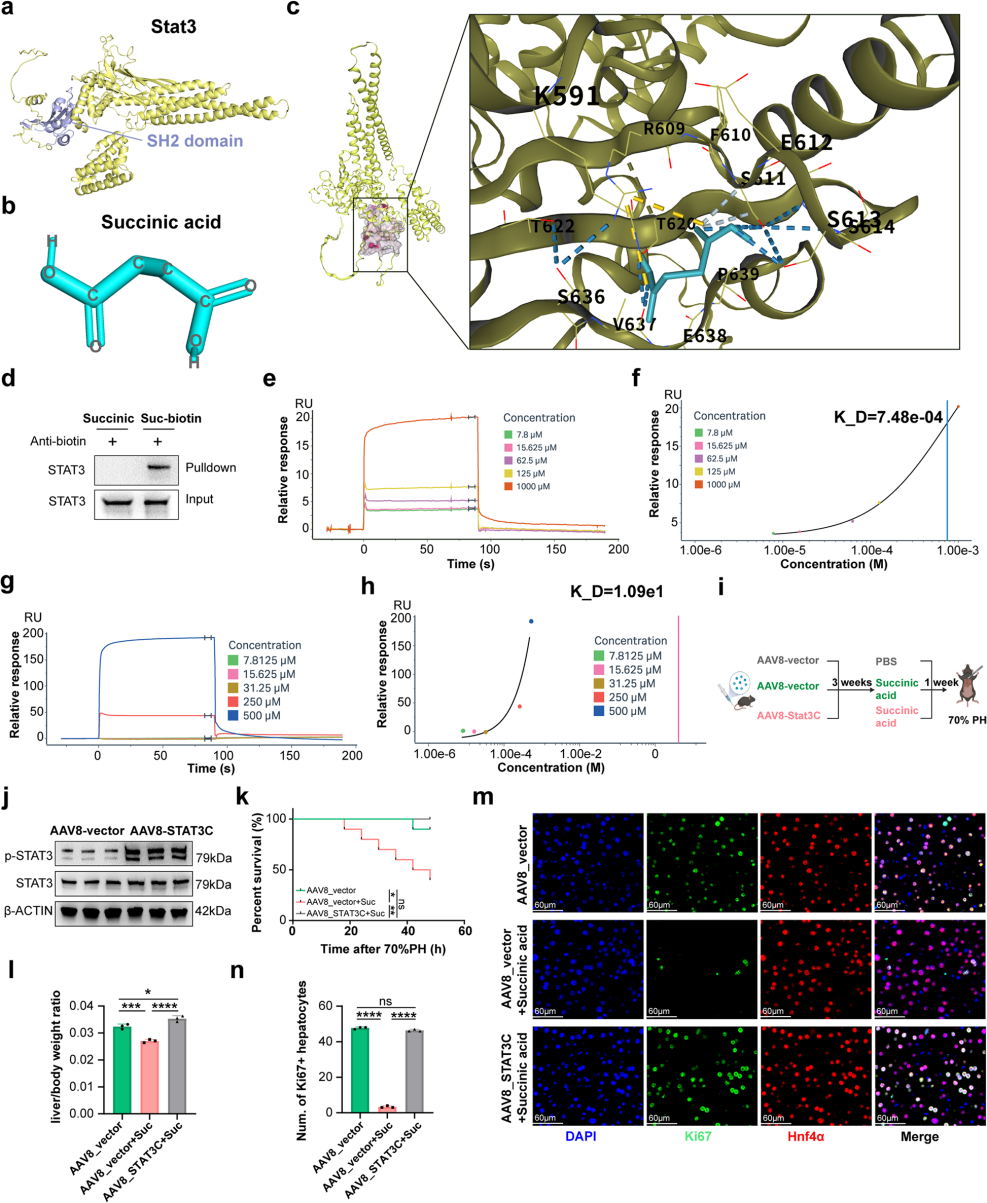
Evidence supporting an interaction between succinic acid and the STAT3 SH2 domain in regulating IL-6–STAT3 signaling. **a** The three-dimensional structure of the mouse full-length STAT3 protein (violet: SH2 domain). **b** The chemical structure of succinic acid (C_4_H_6_O_4_). **c** Molecular docking analysis showing the predicted binding pocket of succinic acid within the STAT3 SH2 domain. Succinic acid interacts with the SH2 domain through hydrogen bonds, weak hydrogen bonds, and ionic interactions. Blue dashed lines indicate hydrogen bonds, light blue dashed lines indicate weak hydrogen bonds, and yellow dashed lines indicate ionic interactions. **d** Biotin pull-down assay showing the interaction between biotinylated succinic acid (Suc-biotin) and STAT3 protein. Anti-biotin antibody was used for pull-down analysis. (*n* = 3). **e** Surface plasmon resonance (SPR) assay of succinic acid binding to the full-length mouse STAT3 protein at different concentrations—the sensorgram of succinic acid binding to the STAT3-immobilized chip. **f** Steady-state affinity fitting curve derived from SPR analysis of succinic acid binding to full-length STAT3 protein. The calculated dissociation constant (K_D) is 7.48 × 10e − 4 M. **g** SPR sensorgrams showing the interaction between succinic acid and mutant STAT3 protein containing SH2-domain mutations predicted from molecular docking analysis. **h** Steady-state affinity fitting curve derived from SPR analysis of succinic acid binding to mutant STAT3 protein. The calculated K_D is 1.09 × 10e1 M. **i** Schematic illustration of the in vivo experimental design. Mice received a tail vein injection of AAV8-vector or AAV8-STAT3C three weeks before succinic acid treatment. Succinic acid was administered for 1 week before 70% partial hepatectomy (PH). **j** Representative western blot analysis confirming constitutive STAT3 activation in mouse liver tissues following AAV8-STAT3C administration. Protein levels of phosphorylated and total STAT3 were analyzed. β-actin served as a loading control. (*n* = 3). **k** Kaplan–Meier survival curves of mice after 70% PH under the indicated treatment conditions. Statistical significance was analyzed using the log-rank test. (*n* = 10). **l** Liver-to-body weight ratio at 48 h after 70% PH in the indicated groups. (*n* = 3). **m** Representative immunofluorescence staining of Ki67 (green) and Hnf4α (red) in liver sections from the indicated groups at 48 h after PH. Nuclei were counterstained with DAPI (blue). Scale bars, 60 μm. (*n* = 3). **n** Quantification of Ki67-positive hepatocytes in liver sections shown in (m). (*n* = 3). Data are presented as mean ± SEM. Statistical significance was determined using one-way ANOVA followed by multiple-comparison testing. ns, not significant; *P < 0.05; **P < 0.01; ***P < 0.001; ****P < 0.0001

To investigate the molecular mechanism by which succinic acid suppresses hepatocyte STAT3 signaling, we performed molecular docking and binding analyses to evaluate the interaction between succinic acid and STAT3. The predicted structure of full-length mouse STAT3 and the chemical structure of succinic acid are presented in Fig. 7a–b, respectively. Structural analysis suggested a potential succinic acid–binding pocket within the STAT3 SH2 domain. Molecular docking predicted that succinic acid interacts with the STAT3 SH2 domain through multiple non-covalent interactions, including hydrogen bonds, weak hydrogen bonds, and ionic interactions (Fig. 7a–c). In the interaction model, blue indicates hydrogen bonds, light blue indicates weak hydrogen bonds, and yellow indicates ionic interactions. To experimentally validate this interaction, we performed biotin pull-down assays using biotinylated succinic acid, which successfully enriched STAT3 protein (Fig. 7d), and surface plasmon resonance (SPR) assays further supported an interaction between succinic acid and full-length STAT3, with an estimated dissociation constant (K_D) of 7.48 × 10E-4 M (Fig. 7e–f). In contrast, mutation of the predicted SH2-domain binding region markedly reduced the binding affinity between succinic acid and STAT3 (Fig. 7g–h), supporting the proposed binding interface.

To evaluate whether STAT3 signaling contributes functionally to succinic acid-mediated impairment of liver regeneration, we generated an AAV8-TBG-STAT3C virus to achieve hepatocyte-restricted constitutive activation of STAT3. STAT3C harbors A662C and N664C substitutions, which promote spontaneous STAT3 dimerization and sustained transcriptional activity independent of upstream cytokine signaling (Fig. 7i). Efficient hepatocyte-targeted activation of STAT3 by AAV8-STAT3C was confirmed by western blot analysis of STAT3 phosphorylation in liver tissues (Fig. 7j). Functionally, constitutive STAT3 activation partially rescued the succinic acid-induced regenerative impairment after 70% PH. AAV8-STAT3C significantly improved post-hepatectomy survival compared with succinic acid-treated controls (Fig. 7k) and restored the liver-to-body weight ratio (Fig. 7l). Moreover, Ki67 immunostaining demonstrated a substantial increase in hepatocyte proliferation in the AAV8-STAT3C group, indicating that constitutive STAT3 activation alleviated succinic acid-induced impairment of liver regeneration (Fig. 7m–n).

To further validate the interaction between succinic acid and the predicted STAT3 SH2 binding pocket, a competitive binding assay was performed using a synthetic peptide spanning the predicted binding region and its flanking amino acids within the STAT3 SH2 domain (15–25 aa) (Fig. S4a). Succinic acid inhibited IL-6-induced STAT3 phosphorylation in hepatocytes, whereas competing peptide treatment restored STAT3 activation, further supporting the proposed interaction of succinic acid with the STAT3 SH2 domain (Fig. S4b–c).

Together, these findings support an interaction between succinic acid and the STAT3 SH2 domain and suggest that this interaction may contribute to reduced hepatocyte STAT3 signaling and impaired liver regeneration.

## Discussion

Liver regeneration is a highly coordinated process that requires metabolic adaptation, inflammatory signaling, and hepatocyte proliferation [31]. Although metabolic remodeling is essential for regeneration after liver injury or partial hepatectomy, the role of individual TCA cycle metabolites remains incompletely understood. In this study, we investigated the relationship between metabolism and cell proliferation using a combination of clinical multi-omics analyses, radiomics, animal models, and mechanistic experiments.

We confirmed that TCA cycle activity is pivotal for liver regeneration (Figs. 1, 2, 3), consistent with studies linking mitochondrial metabolism to hepatocyte proliferation [32]. After partial hepatectomy, the remaining hepatocytes undergo extensive proliferation, leading to a reduction in the number of functional liver cells and impaired lipid metabolism [33]. Hepatocyte damage after PH may lead to the release of succinic acid. Succinic acid not only serves as a metabolic intermediate but also acts as a signaling molecule to regulate gene expression.

Our study identified a dual role of succinic acid in liver regeneration, revealing distinct cell type–specific effects on macrophages and hepatocytes. The potential mechanisms underlying these differential responses warrant further clarification. Hepatocytes provide most of the hepatic functions related to body homeostasis and account for more than 80% of liver mass (approximately 60–70% of all liver cells and account for approximately 80% of liver mass/volume) [34, 35]. In contrast, hepatic macrophages represent a smaller cellular population (approximately 5–10% of total liver cells) but play a critical regulatory role in liver regeneration through secretion of cytokines, including IL-6, which initiate and coordinate regenerative responses [19, 36]. In macrophages, succinate primarily functioned as an immunometabolism signaling molecule. Previous studies have suggested that metabolic stress-induced extracellular succinate accumulation activates the SUCNR1 (GPR91) receptor in immune cells, promoting inflammatory signaling and the production of pro-inflammatory cytokines, including IL-6, IL-1β, and TNF-α [37]. Activation of this pathway has been associated with macrophage inflammatory responses and hepatic stellate cell activation, which may contribute to liver inflammation and fibrosis [25, 38].

In our study, succinate treatment enhanced IFN-γ-induced JAK2/STAT1 activation and increased pro-inflammatory cytokine expression in macrophages, and these effects were partially attenuated by SUCNR1 inhibition, supporting the involvement of receptor-mediated signaling in macrophage activation. In contrast, hepatocytes exhibited a distinct response pattern to succinate. As the major cellular component responsible for restoring liver mass, hepatocytes require tightly regulated proliferative signaling during regeneration. Succinate accumulation may influence hepatocyte function through intracellular metabolic regulation following uptake via dicarboxylate transporters such as NaDC3/SLC13A3, rather than inducing inflammatory activation [39]. In our study, succinate directly impaired IL-6-induced STAT3 phosphorylation in hepatocytes, reduced the expression of regeneration-associated genes, and suppressed hepatocyte proliferation. Furthermore, our biochemical analyses indicated that succinate could interact with the STAT3 SH2 domain, providing a potential mechanism by which a TCA cycle metabolite may influence regenerative signaling through modulation of the STAT3 pathway.

Our study has several limitations. First, the clinical cohort size was limited, and larger cohorts are needed to validate the association between succinate accumulation and liver regeneration outcomes. Second, although our data support an interaction between succinate and STAT3, the structural basis requires further validation. Third, although citrate was also elevated in patients with poor regeneration and showed inhibitory effects, we focused on succinate due to its stronger multi-omics association and established signaling role; whether citrate contributes independently or synergistically remains unclear [40–42]. In addition, the exogenous succinate dose in mice was based on previous rodent studies rather than patient levels, and its dose–response relationship and translational relevance require further investigation. Finally, in vitro models cannot fully reproduce the injured liver microenvironment, and the cell type–specific distribution and metabolic fate of succinate warrant further study using approaches such as metabolic tracing and spatial metabolomics.

Collectively, our findings highlight the importance of metabolic regulation in liver regeneration and suggest that succinate accumulation may represent a maladaptive metabolic response associated with impaired regenerative capacity. Further studies are required to determine whether modulation of succinate metabolism can improve regenerative outcomes in clinical settings.

## Conclusion

In summary, our study suggests that succinic acid may act as an immunometabolic regulator of liver regeneration. Multi-omics analyses identified succinate accumulation in poorly regenerative liver tissues after split liver transplantation, and functional studies demonstrated that elevated succinate was associated with impaired hepatocyte proliferation and liver regeneration following partial hepatectomy. Mechanistically, succinate exhibited cell type–specific effects, promoting macrophage inflammatory activation while attenuating hepatocyte IL-6–STAT3 signaling. Structural and biochemical analyses suggested a potential interaction between succinate and the STAT3 SH2 domain, which may contribute to impaired regenerative signaling. Furthermore, constitutive activation of STAT3 partially rescued succinate-associated regenerative defects in vivo. Together, these findings reveal a potential link between TCA cycle remodeling and liver regeneration failure and highlight the succinate pathway as a candidate target for future therapeutic investigation.

## Method and materials

### Patients and clinical sample collection

Patients who underwent split liver transplantation (SLT) at Shulan (Hangzhou) Hospital between 2021 and 2022 were retrospectively screened. Eligible patients were required to have available donor liver graft specimens, complete perioperative clinical data, adequate postoperative imaging for radiomics-based regeneration assessment, and sufficient tissue samples for transcriptomic and metabolomic analyses. Patients were excluded if they had incomplete clinical or follow-up data, inadequate imaging or sample quality, massive intraoperative bleeding (blood loss ≥ 5,000 mL or transfusion of ≥ 10 units of packed red blood cells), or other perioperative conditions unrelated to graft regeneration that could affect evaluation.

Among 55 screened SLT cases, 20 patients were included in the final analysis and stratified into high-regeneration (LR⁺, *n* = 10) and low-regeneration (LR⁻, *n* = 10) groups according to radiomics-derived regeneration rates.

### Human samples

Twenty human liver samples were collected via surgical procedures at Shulan Hospital. These samples were submerged in liquid nitrogen to freeze quickly and stored at −80 °C.

### Liver weight calculation

We used *3D Slicer* to sketch all liver contours on all CT slices and to reconstruct the livers from the CT images [43]. We calculated the liver regeneration rate per day using graft volume (GV; pre-GV) and CT images obtained after surgery (post-GV). Liver regeneration rate (LRR) = [(post-GV-pre-GV)/pre-GV]/days. The liver volume (GV) to standard liver volume (SLV) ratio, calculated as (GV/SLV), was also evaluated to assess liver regeneration capacity (LR). Increased GV/SLV = post-LV/SLV = post LV/SLV**–** pre GV/SLV.

### Animal experiments

Male C57BL/6 mice aged 6–8 weeks were obtained from the Model Animal Research Center of Nanjing University (Nanjing, China) and randomly divided into different treatment groups, with 10 animals in each group. For 70% partial hepatectomy, mice were anesthetized with isoflurane (5% for induction and 3% for maintenance). Approximately 70% of the liver mass, including the left lateral lobe (30%), right median lobe (30%), and left median lobe (10%), was surgically removed. Liver lobes were ligated at the base using pre-positioned silk sutures before resection [44, 45].

### Flow cytometry

Single cells were isolated from the livers of control and PH mice and digested using a modified liver collagenase perfusion method. Total immune cells were purified using Mouse 1 × Lymphocyte Separation Medium (E312E551, DAYOU, Guangdong, China) according to the manufacturer’s instructions. Cells were stained with Fixable Viability Stain 780 (1:1000, 565388, BD Biosciences) to exclude dead cells. Surface staining was subsequently performed in FACS buffer at 4 °C for 60 min using the following antibodies: CD45 (1:100, 157,213, Biolegend, San Diego, CA, USA), CD11b (1:100, 101,245, Biolegend), CD3 (1:100, 100,205, Biolegend), CD20 (1:100, 150,411, BioLegend), F4/80 (1:100, 111,603, BioLegend), CLEC4F (1:100, 156,803, BioLegend), and Ly6C (1:100, 128,017, BioLegend). Flow cytometry acquisition was performed using a BD FACSCanto™ flow cytometer, and data were analyzed with FlowJo v10 software.

### AAV8-mediated *Suclg1* knockdown in the liver

To reduce hepatic succinate production in vivo, an AAV8-mediated shRNA strategy was used to knock down *Suclg1*, a key subunit of succinyl-CoA synthetase involved in succinate generation. shRNA sequences targeting mouse *Suclg1* or a scrambled negative control were cloned into an AAV8 vector under the control of the U6 promoter, generating AAV8-shNC and AAV8-sh*Suclg1* constructs. Recombinant AAV8 particles were produced, purified, and titrated as viral genomes (vg)/mL. Mice were randomly assigned to the AAV8-shNC, AAV8-sh*Suclg1*, or AAV8-sh*Suclg1* plus succinic acid groups. AAV8 particles were administered via tail vein injection at a dose of 2 × 10^11 vg per mouse in 100**–**200 μL sterile PBS. Two to three weeks after viral delivery, mice underwent 70% PH. In the rescue group, succinic acid was administered to restore succinate exposure. Liver tissues were collected at the indicated time points for analysis of *Suclg1* knockdown efficiency, hepatic succinate levels, STAT3 activation, and hepatocyte proliferation.

### AAV8-mediated hepatocyte-specific constitutive activation of STAT3

To achieve hepatocyte-specific constitutive activation of STAT3 in vivo, the coding sequence of constitutively active STAT3 (STAT3C), containing the A662C and N664C substitutions, was cloned into an AAV8 vector under the control of the hepatocyte-specific thyroxine-binding globulin (TBG) promoter. The resulting constructs were designated AAV8-TBG-STAT3C and AAV8-TBG-vector.

Recombinant AAV8 particles were produced, purified, and titrated as viral genomes (vg)/mL. Mice received AAV8-TBG-vector or AAV8-TBG-STAT3C via tail vein injection at a dose of 2 × 10^11 vg per mouse in 100**–**200 μL sterile PBS. Two to three weeks after viral delivery, mice underwent succinic acid treatment followed by 70% partial hepatectomy. Liver tissues were collected at the indicated time points for analysis of STAT3 phosphorylation, liver regeneration, and hepatocyte proliferation.

### Molecular docking

We used DockThor, a protein–ligand docking software, to predict the binding modes and relative binding energies between mouse STAT3 and succinic acid. The molecular structure of succinic acid was obtained from PubChem (https://pubchem.ncbi.nlm.nih.gov/) [46]. The predicted three-dimensional structure of mouse STAT3 was obtained from the AlphaFold Protein Structure Database (AlphaFold model ID: AF-P42227-F1, corresponding to UniProt accession P42227). The protein structure was prepared for docking by removing water molecules and adding polar hydrogen atoms, followed by conversion to PDBQT format. Molecular docking was performed using the DockThor web server (https://dockthor.lncc.br/), which employs a validated grid-based algorithm with hybrid scoring functions. The docking grid was centered on the SH2 domain region of STAT3 to investigate potential succinic acid binding. The docking region was defined as a 20 Å × 20 Å × 20 Å cubic grid centered on the SH2 domain according to the reported functional domain architecture of STAT3. A genetic algorithm with 100 runs per ligand, a population size of 150, and a maximum of 25,000 iterations was applied. DockThorScore integrates van der Waals, electrostatic, and desolvation energy terms. The top-ranked docking poses were selected based on predicted binding energy (ΔG ≤ − 8.0 kcal/mol) and ligand RMSD (< 2.0 Å). PyMOL v2.5.4 (Schrödinger) was used for structural visualization, hydrogen bond analysis, and preparation of publication-quality figures. Key interactions, including hydrogen bonds and salt bridges, were annotated based on distance criteria (< 3.5 Å for hydrogen bonds).

### Surface plasmon resonance (SPR)

The binding interaction between purified recombinant proteins and succinic acid was assessed by surface plasmon resonance (SPR) using a Biacore™ 1K system (Cytiva). The full-length mouse STAT3 protein (CSB-YP022812MO, Cusabio, Wuhan, China) was captured on a Series S CM5 chip (Cytiva) via chelation at pH 4.5. The running buffer used was HBS-EP + (10 × HBS-EP + , Cytiva). Serially diluted succinic acid samples prepared in running buffer were injected onto the sensor chip, and SPR measurements were performed at 25 °C with a flow rate of 30 μL/min. To validate the predicted binding interface identified by molecular docking, full-length STAT3 SH2-domain mutant proteins were designed based on the predicted binding residues (synthesized and purified by Sanyou Biopharmaceuticals Co., Ltd, Shanghai, China). SPR assays between mutant STAT3 proteins and succinic acid were subsequently performed under the same experimental conditions. K_D values were derived from the binding kinetics analysis using Biacore™ 1K Insight Evaluation Software.

### Biotin–succinic acid pull-down assay

Biotin-labeled succinic acid was synthesized by randomly coupling biotin to one of the two carboxyl groups of succinic acid. Cell lysates were incubated with biotin–succinic acid at 4 °C, while unconjugated succinic acid served as the control. Streptavidin magnetic beads were then added to capture biotin-labeled complexes. After washing, bound proteins were eluted with SDS loading buffer and analyzed by western blotting using an anti-STAT3 antibody. Input samples were analyzed in parallel to confirm equal protein loading.

### Competitive peptide assay

The competitive peptide was designed based on the predicted succinic acid-binding region within the STAT3 SH2 domain. The peptide was chemically synthesized by standard solid-phase peptide synthesis using the Fmoc strategy. After synthesis, the crude peptide was cleaved from the resin, deprotected, and purified by reverse-phase high-performance liquid chromatography. Peptide identity and molecular weight were confirmed by mass spectrometry, and purity was > 95%. Primary hepatocytes were pretreated with succinic acid, with or without the competing peptide, and then stimulated with IL-6 (10 ng/mL) for 30 min. Cells were collected and lysed for Western blot analysis. STAT3 activation was evaluated by detecting p-STAT3 and total STAT3 levels, with β-actin used as the loading control. The relative p-STAT3/STAT3 ratio was quantified to assess whether the peptide rescued succinic acid–mediated inhibition of IL-6–STAT3 signaling.

### Statistical analysis

No data preprocessing was performed. Mice were randomly assigned to groups (*n* = 10 per group), and deceased animals were excluded from further analysis. Immunohistochemical and HE staining were performed in a blinded manner, and liver sections were quantified using ImageJ 1.53t, with each sample analyzed at least three times. Statistical analyses were conducted using GraphPad Prism 8.0, and data are shown as mean ± SD. IHC, flow cytometry, and qPCR assays were independently repeated at least three times. Data normality was assessed using the Shapiro–Wilk test. Comparisons between two groups were performed using an unpaired two-tailed Student’s t-test, while one-way ANOVA was applied for multiple groups with normally distributed data. Survival curves were compared using the log-rank test. *P* < 0.05 was considered statistically significant (* *P* < 0.05).

Detailed experimental procedures are described in the Supplementary Methods.

## Supplementary Information

Supplementary Material 1.

## Data Availability

The transcriptomic sequencing data have been deposited in the NCBI Gene Expression Omnibus (GEO) under accession number GSE331120.
