# Supplementary material for: Succinic acid inhibits hepatocyte regenerative signaling via STAT3 SH2-domain engagement
Source: Mol Biomed. 2026 Aug 8;7:132. doi: 10.1186/s43556-026-00535-z (PMC13451177; doi:10.1186/s43556-026-00535-z)
Supplement: Supplementary file 1 [file 43556_2026_535_MOESM1_ESM.docx]

**Succinic acid inhibits hepatocyte regenerative signaling via STAT3 SH2-domain engagement**

**Supplementary Figure 1-4**

**Figure S1 Time-series clustering analysis reveals distinct transcriptional programs associated with impaired liver regeneration in diabetes and HBV cohorts**

a. Time-series clustering analysis of differentially expressed genes in diabetes-associated liver regeneration groups (Diabetes^+^LR^+^, Diabetes^+^LR^−^, Diabetes^−^LR^+^, and Diabetes^−^LR^−^). Heatmaps show clustered gene expression patterns with corresponding temporal trends, and representative enriched KEGG pathways for each cluster. Color intensity represents normalized Z-scores of gene expression.
b. Time-series clustering analysis of differentially expressed genes in HBV-associated liver regeneration groups (HBV^+^LR^+^, HBV^+^LR^−^, HBV^−^LR^+^, and HBV^−^LR^−^). Heatmaps display clustered gene expression dynamics, and representative enriched KEGG pathways associated with each cluster. Color intensity represents normalized Z-scores of gene expression.

**Figure S2 Dynamic expression of TCA cycle-related genes during liver regeneration after partial hepatectomy**

a. Uniform manifold approximation and projection (UMAP) plot of 435,413 single cells collected at different time points after 70% partial hepatectomy (8 h, D0, D1, D2, D3, and D7). Cell populations are annotated by cell type (left) and time point (right).
b. Feature plots showing the expression patterns of representative TCA cycle-related genes, including *Idh1*, *Idh3b*, *Suclg1*, *Suclg2*, *Sdha*, *Sdhb*, *Sdhc*, and *Sdhd*, across liver cell populations during regeneration.
c. Heatmap showing the dynamic expression changes of TCA cycle-related genes at different time points after 70% PH. Color intensity indicates relative gene expression levels.
d. qRT–PCR analysis of liver regeneration-associated genes, including *MKi67*, *Pcna*, *Ccnd1*, *Cdk4*, and *Sox9*, in liver tissues collected 48 h after 70% PH from mice treated with PBS, citric acid, or succinic acid. The genes were normalized to GAPDH mRNA levels in each sample. (Pcna: Proliferating cell nuclear antigen; *MKi67*: marker of proliferation Ki-67; *Sox9*: Sex determining region Y)-box 9; *Cdk4*: Cyclin dependent kinase 4; *Ccnd1: Cyclin D1*). (n=3)

Data are presented as mean ± SEM. Statistical significance was determined using two-way ANOVA followed by multiple-comparison testing. ns, not significant; **P* < 0.05; ***P* < 0.01; ****P* < 0.001; *****P* < 0.0001.

**Figure S3 Succinate promotes macrophage inflammatory activation and inhibits hepatocyte STAT3 signaling through macrophage-derived paracrine factors**

a. Schematic illustration of the experimental design. Macrophages were treated with succinic acid (40 μg/mL) in the presence or absence of the SUCNR1 inhibitor (5 μM) for 24 h, followed by IFN-γ stimulation (10 ng/mL) for 30 min.
b. ELISA quantification of TNF-α, IL-1β, and IL-6 levels in macrophage culture supernatants under the indicated conditions. (n=3)
c. Representative western blot analysis of JAK–STAT signaling pathway activation in macrophages treated with succinic acid and/or SUCNR1 inhibitor. Protein levels of phosphorylated and total JAK1, JAK2, STAT1, and STAT3 were analyzed. β-actin served as a loading control. (n=3)
d. Quantification of protein expression levels shown in (c). Relative phosphorylation levels were normalized to the corresponding total protein levels. (n=3)
e. qRT–PCR analysis of inflammatory cytokine gene expression, including *Il1β*, *Il6*, and *Tnf*, in macrophages under the indicated conditions. (n=3)
f. Schematic illustration of the macrophage–hepatocyte conditioned medium (CM) experiment. Supernatants collected from control or succinate-treated macrophages were incubated with or without IL-6 neutralizing antibody before treatment of primary hepatocytes.
g. Representative western blot images of STAT3 signaling and downstream cell-cycle regulators in hepatocytes treated with macrophage-derived conditioned medium. Neutralization of IL-6 partially restored STAT3 phosphorylation and the expression of regenerative markers, including CCND1, CCNE1, and CDK1. (n=3)
h. Quantification of protein expression levels shown in (g). Relative phosphorylation levels were normalized to the corresponding total protein levels. (n=3)

Data are presented as mean ± SEM. Statistical significance was determined using one-way ANOVA or two-way ANOVA followed by multiple-comparison testing. ns, not significant; **P* < 0.05; ***P* < 0.01; ****P* < 0.001; *****P* < 0.0001.

**Figure S4 Competitive peptide assay validating succinic acid binding to the STAT3 SH2 domain**

a. Schematic illustration of the competitive binding assay. A synthetic peptide spanning the predicted STAT3 SH2-binding region was used to competitively block succinic acid binding in primary hepatocytes, followed by IL-6 stimulation.
b. Representative western blot analysis of phosphorylated and total STAT3 in hepatocytes treated with succinic acid in the presence or absence of the competing peptide. β-actin served as a loading control. (n=3)
c. Quantification of STAT3 phosphorylation levels shown in (B). Relative protein expression was normalized to total STAT3. (n=3)

Data are presented as mean ± SEM. Statistical significance was determined using one-way ANOVA followed by multiple-comparison testing. ns, not significant; *****P* < 0.0001.

**Supplementary methods**

**RNA Sequencing Analysis**

Total RNA was isolated using TRIzol® Reagent (Invitrogen), followed by DNase I treatment to eliminate genomic DNA contamination. RNA integrity was assessed using an Agilent 2100 Bioanalyzer, with all samples achieving an RNA Integrity Number (RIN) of ≥ 8. Stranded mRNA-seq libraries were constructed using the NEBNext® Ultra™ II Directional RNA Library Prep Kit (NEB) according to the manufacturer's protocol. RNA quantification was performed using NanoDrop™ 2000 (Thermo Fisher Scientific, NY, USA). Raw reads were processed by quality control, alignment, and quantification. RNA-seq data for human liver tissue samples were mapped to the GRCh38 (Ensembl 93) reference genome using STAR, and gene expression was quantified using an RSEM-based pipeline. DEGs were determined using DESeq2 (v.1.30.1) and were defined as having an absolute fold change of >1 and a P-value of <0.05.

**scRNA-seq analysis**

All raw scRNA-seq data were downloaded from the GEO database (GSE254481). All processed data can be downloaded and visualized on the LISTA website (db.cngb.org/stomics/lista)^29^.

**Metabolomics analysis**

Data were processed using principal component analysis (PCA) to visualize group differences and to assess model reliability through permutation tests. DEGs were determined using DESeq2 (v.1.30.1), with statistical significance set at (*P* < 0.05). KEGG pathway enrichment analysis was conducted using the identified differential metabolites to elucidate their involvement in metabolic pathways. The results were visualized using bubble plots to highlight enriched pathways and their associated metabolites.

**Transcriptomic and Metabolomic Correlation Analysis**

Metabolite and mRNA levels were log-transformed and scaled to a mean of zero and a standard deviation of 1. Pearson’s correlation coefficients were calculated for each pairwise mRNA-metabolite combination within each study and meta-analyzed across studies using the metacor function in the General Package for Meta-Analysis in R. A correlation heatmap was generated using the Complex Heatmap R package, in which the organization of metabolites was fixed by the Reference Metabolite Database superclass, main class, and subclass. Genes were allowed to self-organize using the default complete linkage method of the hierarchical clustering function.

**Identification of gene co-expression modules and association with clinical traits**

Weighted gene co-expression network analysis (WGCNA) was performed using the WGCNA R package to identify gene networks related to liver regeneration. Briefly, pairwise Pearson correlations among genes were calculated to generate a similarity matrix, which was converted into an adjacency matrix and subsequently a topological overlap matrix. The soft-thresholding power was determined based on the scale-free network topology, and a value of 18 was selected to achieve a scale-free topology fit index of 0.8. Genes were hierarchically clustered based on their co-expression patterns, and distinct modules were generated from the resulting dendrogram. The relationship between each module and clinical traits was evaluated, and modules significantly associated with regeneration-related phenotypes (P < 0.05) were considered clinically relevant and subsequently analyzed.

**Cell culture**

Primary peritoneal macrophages isolated from mice were cultured in RPMI-1640 supplemented with 10% fetal bovine serum (FBS, Gibco, Thermo Fisher Scientific) and 1% penicillin–streptomycin. Inflammatory activation was induced by exposure to IFN-γ (10 ng/mL) for 30 min.

Primary hepatocytes were isolated from mouse livers using a modified collagenase perfusion protocol. Briefly, liver tissues were perfused with Hank’s balanced salt solution (HBSS, H4641, Sigma-Aldrich, MO, USA) at a flow rate of 5 mL/min, followed by digestion with 0.025% collagenase solution. Cell viability was assessed by trypan blue staining, and samples with viability >60% were used for downstream experiments.

Freshly isolated hepatocytes were cultured in William's E medium (12551032, Thermo Fisher Scientific) supplemented with 10% FBS and 1% penicillin–streptomycin. Following a 4 h attachment period, cells were transferred to fresh medium without FBS and stimulated with IL-6 (10 ng/mL) for 30 min before analysis. Mouse IL-6 neutralizing antibody (BE0046, Bio X Cell, NH, USA) was used to block IL-6 activity according to the manufacturer's instructions.

**Western blotting**

Total proteins were extracted using ice-cold RIPA buffer (9803, Cell Signaling Technology, CST, MA, USA) supplemented with protease and phosphatase inhibitor cocktails (5872, CST). Protein concentrations were determined using a BCA assay kit (23227, Thermo Fisher Scientific). Equal amounts of protein were mixed with 5× loading buffer (P0286, Beyotime, Jiangsu, China), denatured at 95 °C for 15 min, separated by 10% SDS–PAGE, and transferred onto PVDF membranes (IPVH00010, Millipore, MA, USA).

Membranes were blocked and incubated with primary antibodies overnight at 4 °C, then treated with HRP-conjugated secondary antibodies (35401, 1:2000; CST) for 1 h at room temperature. Immunoreactive signals were detected using enhanced chemiluminescence substrate (32106, Thermo Fisher Scientific). Densitometric quantification was performed with β-actin serving as the internal loading control.

Primary antibodies included anti-STAT1 (1:1000, 14994, CST ), anti-phospho-STAT1 (9167, 1:1000, CST), anti-STAT3 (4904, 1:1000, CST), anti-phospho-STAT3 (9145, 1:1000, CST ), anti-JAK1 ( 3344, 1:1000, CST ), anti-phospho-JAK1 (3331, 1:1000, CST ), anti-JAK2 (3230, 1:1000, CST), anti-phospho-JAK2 (3776, 1:1000, CST), and anti-β-Actin (4967, 1:2000, CST ).

**Quantitative real-time PCR**

Total RNA was isolated using TRIzol Reagent (Invitrogen), followed by reverse transcription into complementary DNA (cDNA) using the PrimeScript RT Master Mix kit (11150ES60, YESEN, Shanghai, China). qRT-PCR assays were conducted on a Q6 Real-Time PCR System (Applied Biosystems, Thermo Fisher Scientific) using SYBR Green Master Mix (11201ES08, YESEN). Relative mRNA expression levels of target genes were calculated after normalization to GAPDH. Three independent biological replicates were included for each experimental group, and each sample was analyzed in triplicate technical repeats.

The primers used for qRT-PCR were as follows:

| Gene name | Forward primer (5′-3′) | Reserve primer (5′-3′) |
| --- | --- | --- |
| *GAPDH* | AGGTCGGTGTGAACGGATTTG | TGTAGACCATGTAGTTGAGGTCA |
| *Sox9* | GAGCCGGATCTGAAGAGGGA | GCTTGACGTGTGGCTTGTTC |
| *MKi67* | GAGGAGAAACGCCAACCAAGAG | TTTGTCCTCGGTGGCGTTATCC |
| *Pcna* | TTTGAGGCACGCCTGATCC | GGAGACGTGAGACGAGTCCAT |
| *Ccnd1* | GCGTACCCTGACACCAATCTC | CTCCTCTTCGCACTTCTGCTC |
| *Hgf* | GGTGACCAAACTCCTGACAG | CAGGCTGGTGTTGTCATGTC |
| *Cdk4* | ATGGCTGCCACTCGATATGAA | TCCTCCATTAGGAACTCTCACAC |
| *Lrp-5* | AAGGGTGCTGTGTACTGGAC | AGAAGAGAACCTTACGGGACG |
| *Lrp-6* | TTGTTGCTTTATGCAAACAGACG | GTTCGTTTAATGGCTTCTTCGC |
| *IL-6*: | ATGGCGTTACTGGATCTGTGC | CGCGGAGAAACTGTAGTGTCC |
| *IL-1b* | GCAACTGTTCCTGAACTCAACT | ATCTTTTGGGGTCCGTCAACT |
| *Tnf* | CCCTCACACTCAGATCATCTTCT | GCTACGACGTGGGCTACAG |

**Immunohistochemical staining**

The liver tissues were embedded, sliced (4 µm thick), dried, dewaxed, and rehydrated for the experiments. Anti-Ki67 antibody (1:200, ab15580, Abcam) and anti-Hnf4α antibody (1:200, 3113, CST) were incubated overnight at 4 °C. 488-Goat Anti-Mouse Recombinant Secondary Antibody or 555-Goat Anti-Rabbit Recombinant Secondary Antibody (1:100, RGAM002, RGAG003, Proteintech, Wuhan, China) were used as secondary antibodies. DAPI (ab104139; Abcam, Cambridge, UK) was used for nuclear staining. Slides were mounted and visualized using an OLYMPUS (FV3000) microscope.

**Serum analysis**

Mouse serum alanine aminotransferase (ALT, E-BC-K235-M, E-lab Science, Jiangsu, China), aspartate aminotransferase (AST, E-BC-K236-M, E-lab Science), mouse IL-6 (E-EL-M0044, E-lab Science), TGF-β (E-EL-M0051, E-lab Science), TNF-α (E-EL-M3063, E-lab Science), IL-1β (E-EL-M0037, E-lab Science), and Succinic acid (E-BC-K902-M, E-lab Science) were measured using an ELISA kit according to the manufacturer's instructions.
